# A high tibial slope, allograft use, and poor patient-reported outcome scores are associated with multiple ACL graft failures

**DOI:** 10.1007/s00167-021-06460-8

**Published:** 2021-01-31

**Authors:** Philipp W. Winkler, Nyaluma N. Wagala, Jonathan D. Hughes, Bryson P. Lesniak, Volker Musahl

**Affiliations:** 1 Department of Orthopaedic Surgery, UPMC Freddie Fu Sports Medicine Center, University of Pittsburgh, 3200 S. Water St, Pittsburgh, PA 15203 USA; 2 Department for Orthopaedic Sports Medicine, Klinikum rechts der Isar, Technical University of Munich, Ismaninger Str. 22, 81675 Munich, Germany

**Keywords:** ACL, Anterior cruciate ligament, Failure, Revision, Quadriceps tendon, Allograft, Tibial slope

## Abstract

**Purpose:**

To compare clinical outcomes, radiographic characteristics, and surgical factors between patients with single and multiple anterior cruciate ligament (ACL) graft failures. It was hypothesized that patients experiencing multiple ACL graft failures exhibit lower patient-reported outcome scores (PROs) and a higher (steeper) posterior tibial slope (PTS) than patients with single ACL graft failure.

**Methods:**

Patients undergoing revision ACL reconstruction with a minimum follow-up of 12 months were included in this retrospective cohort study. Based on the number of ACL graft failures, patients were assigned either to the group “single ACL graft failure “or” multiple ACL graft failures “. The PTS was measured on strict lateral radiographs. Validated PROs including the International Knee Documentation Committee (IKDC) subjective knee form, Knee Injury and Osteoarthritis Outcome Score, Lysholm Score, Tegner Activity Scale, ACL-Return to Sport after Injury Scale, and Visual Analogue Scale for pain were collected.

**Results:**

Overall, 102 patients were included with 58 patients assigned to the single ACL graft failure group and 44 patients to the multiple ACL graft failures group. Quadriceps tendon autograft was used significantly more often (55% vs. 11%, *p* < 0.001) and allografts were used significantly less often (31% vs. 66%, *p* < 0.001) as the graft for first revision ACL reconstruction in patients with single versus multiple ACL graft failures. Patients with multiple ACL graft failures were associated with statistically significantly worse PROs (IKDC: 61.7 ± 19.3 vs. 77.4 ± 16.8, *p* < 0.05; Tegner Activity Scale: 4 (range, 0–7) vs. 6 (range 2–10), *p* < 0.05), higher PTS (12 ± 3° vs. 9 ± 3°, *p* < 0.001), and higher rates of subsequent surgery (73% vs. 14%, *p* < 0.001) and complications (45% vs. 17%, *p* < 0.05) than patients with single ACL graft failure.

**Conclusion:**

Compared to single ACL graft failure in this study multiple ACL graft failures were associated with worse PROs, higher PTS, and allograft use. During the first revision ACL reconstruction, it is recommended to avoid the use of allografts and to consider slope-reducing osteotomies to avoid multiple ACL graft failures and improve PROs.

**Level of evidence:**

Level 3.

## Introduction

The failure rate after revision anterior cruciate ligament reconstruction (ACL-R) has been reported to be 3–21% [1–5] compared to 3–10% after primary ACL-R [6–11]. Anatomical and patient-related risk factors, technical and biological failures, unappreciated concomitant capsuloligamentous or meniscal injuries, as well as aggravated surgical conditions due to previous interventions have been identified as underlying causes for the increased failure rate [1, 12–15].

Despite a large body of the literature regarding single anterior cruciate ligament (ACL) graft failures, only a few studies emphasizing multiple ACL graft failures have been published [1, 5, 12, 13, 15, 16]. Prior studies have shown that undergoing more than one revision ACL-R is a predictor for worse patient-reported outcomes (PROs) and subsequent graft failures [2, 17]. Additionally, the majority of patients that have sustained multiple ACL graft failures present with meniscal tears and cartilage lesions [5, 12, 13, 15, 16], which are well-known negative predictors for long-term knee function. Failure analyses revealed that an increased posterior tibial slope (PTS), static anterior tibial subluxation, and a deep, elliptically shaped, lateral femoral condyle were associated with multiple ACL graft failures [1, 5, 13]. Consequently, a failure rate of up to 30% after a second revision ACL-R was demonstrated [5]. Additionally, it has been shown that patients undergoing more than two revision ACL-Rs are almost 26 times more likely to experience subsequent ACL graft failure than patients undergoing first revision ACL-R [2]. Thus, a vicious circle of multiple ACL graft failures appears to exist in certain patients. Further investigation of the multifactorial etiology of ACL graft failures is warranted to avoid the vicious circle of multiple ACL graft failures. Surgery- and patient-related predictors of multiple ACL graft failures may aid in surgical decision-making and patient counseling for revision ACL reconstruction.

The objectives of this study were to compare clinical outcomes, demographic and radiographic characteristics, and surgical factors between patients with single and multiple ACL graft failures. It was hypothesized that patients experiencing multiple ACL graft failures exhibit lower PROs and a higher (steeper) PTS than patients with single ACL graft failure.

## Materials and methods

This study was approved by the Institutional Review Board of the University of Pittsburgh (No.: STUDY20050226). All patients presenting with single or multiple ACL graft failures in the senior author’s (VM) outpatient clinic between 2010 and 2020 were screened for eligibility for this retrospective cohort study.

**Fig. 1:**
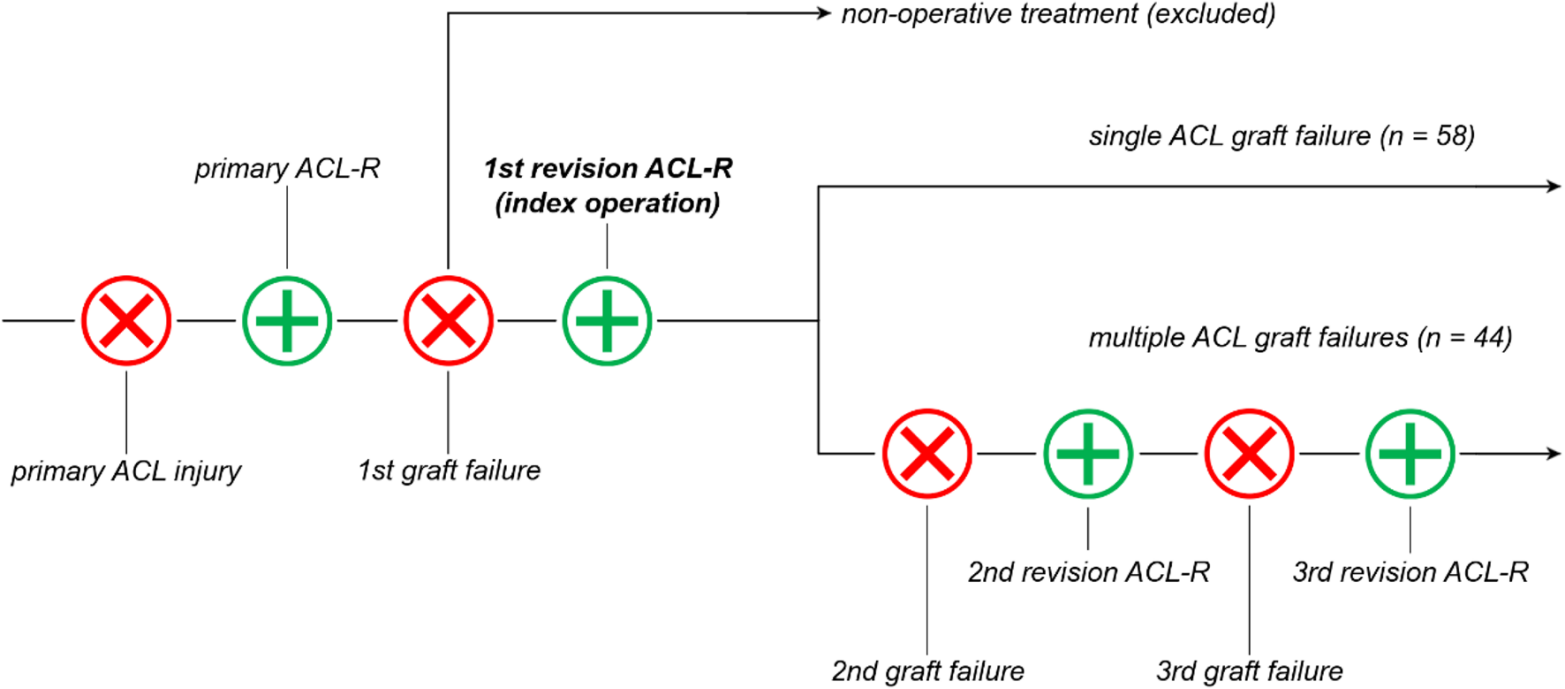
Group allocation based on the number of anterior cruciate ligament graft failures. Encircled “x” illustrating ACL injury. Encircled “+” illustrating ACL reconstruction. ACL, anterior cruciate ligament; ACL-R, ACL reconstruction

Inclusion criteria comprised: single-bundle first revision ACL-R, minimum 12 month follow-up since the first revision ACL-R, available medical records, and anterior–posterior (weight-bearing) and lateral radiographs. Patients with a history of inflammatory arthritis, ipsilateral multiple-ligament knee injuries, or a previous femur or tibia fracture were excluded from this study. Given the need for revision ACL-R to sustain multiple ACL graft failures, the first revision ACL-R was considered as the index operation (Fig. 1). Therefore, patients undergoing non-operative treatment after the first ACL graft failure were excluded from this study.

**Fig. 2:**
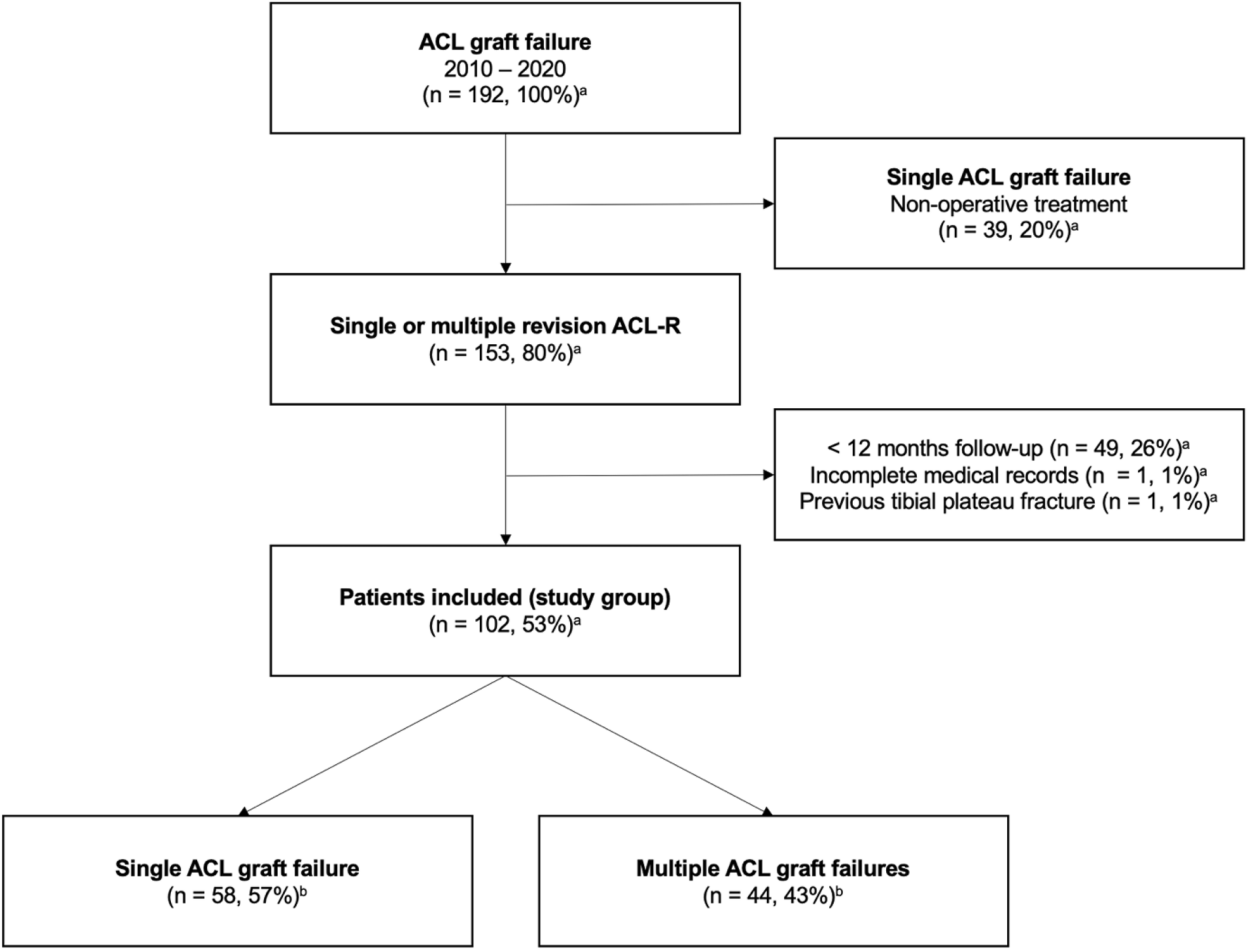
Flowchart of patient enrollment. ^a^Percentage of screened population; ^b^Percentage of total study group; ACL, anterior cruciate ligament; ACL-R, ACL reconstruction

Anterior cruciate ligament graft failure was defined as (1) the need for revision ACL-R due to symptomatic instability, pain, or severe impairment in daily activities, (2) complete ACL graft disruption confirmed by magnetic resonance imaging (MRI) or arthroscopy, or (3) attenuated or partially ruptured graft confirmed by MRI plus side-to-side difference > 5 mm for anterior tibial translation based on KT-1000 (MEDmetric Corp., San Diego, CA, USA) arthrometry. Based on the number of ACL graft failures, the patients were subsequently assigned either to the group "single ACL graft failure" or "multiple ACL graft failures" (Fig. 2).

### Data collection

#### Demographic and surgical data

A comprehensive review of medical records was conducted between March and July 2020 by one observer (PWW). Demographic and surgical data were collected and included the following: sex; laterality; body mass index (BMI); date of birth; date of each ACL injury; date of each ACL-R performed; mechanism of each ACL injury; occurrence, number, and type of complications; occurrence, number, and type of subsequent surgical procedures; concurrently performed surgical procedures during first revision ACL-R; graft choices; and history of contralateral ACL injuries. Atraumatic, traumatic non-contact, and traumatic contact were defined as the three types of injury mechanisms. Complications were categorized and included the following: symptomatic hardware, surgical site infection, meniscus tear requiring reoperation, cartilage lesion requiring reoperation, knee stiffness (10° side-to-side difference in range of motion based on clinical examination), and symptomatic baker cyst confirmed by MRI.

#### Radiographic characteristics

Lateral radiographs with a maximum posterior femoral condyle overlap of 6 mm were used to measure the medial PTS as well as the lateral femoral condyle ratio (LFCR), as previously described in detail [18–21]. Briefly, the medial PTS was defined as the angle between the proximal tibial shaft axis and a tangential line to the medial tibial plateau, subtracted from 90° [18, 21]. To calculate the LFCR, first the distal femoral shaft axis was determined on lateral radiographs. In a second step, the maximum anterior to posterior distance of the lateral femoral condyle (lateral femoral condyle axis) was measured. Next, the distance along the lateral femoral condyle axis between the most posterior part of the lateral condyle and the femoral shaft axis was measured (posterior lateral femoral condyle depth). Subsequently, the posterior lateral femoral condyle depth was divided by the total anterior–posterior length of the lateral femoral condyle to obtain the LFCR [19, 20]. All measurements were performed by observer one (PWW) using Philips iSite PACS (Koninklijke Philips N.V., Amsterdam, NLD), which allows a measurement accuracy of 0.1 mm and 1°, respectively. Intraclass correlation coefficients (ICC) were calculated to assess intra- and interrater reliability for the measurements obtained. Accordingly, the medial PTS and LFCR were measured twice by observer one (PWW; 1-month interval between measurements) and once by observer 2 (NNW) for 20 randomly selected patients. Intrarater ICC of 0.817 (LFCR) and 0.853 (medial PTS) as well as interrater ICC of 0.846 (LFCR) and 0.825 (medial PTS) indicate good reliability of the measurements. Additionally, anterior–posterior weight-bearing radiographs were used to determine the degree of osteoarthritis (OA) based on the Kellgren–Lawrence Scale. The degree of OA was assessed by observers one (PWW) and two (NNW) in agreement with each other.

#### Patient-reported outcome scores (PROs)

Questionnaires including standardized and validated PROs were mailed to all included patients. The following PROs were used for follow-up assessment: International Knee Documentation Committee (IKDC) subjective knee form, Knee Injury and Osteoarthritis Outcome Score (KOOS), Lysholm Score, Tegner Activity Scale, ACL-Return to Sport after Injury Scale (ACL-RSI) [22], and Visual Analogue Scale (VAS) for pain. Written informed consent was obtained from each patient who completed the questionnaire.

### Statistical analysis

A priori power analysis was conducted using the freely available software G*Power (Erdfelder, Faul, Buchner, Lang, HHU Düsseldorf, Düsseldorf, Germany). The PTS is known as a risk factor for ACL graft failure. Therefore, the PTS was considered to be the primary outcome measure and was used for a priori sample size calculation. A recently published study reported a mean PTS of 11.5° ± 3.6° and 13.1° ± 2.4° in patients undergoing revision ACL-R and re-revision ACL-R, respectively [5]. Accordingly, a total sample size of 92 patients (46 patients per group) was required to achieve a statistical power of 0.8 (effect size, 0.52; level of significance, 0.05).

Categorical variables are presented as count and percentage. Normal distribution of the collected continuous variables was assessed by the Shapiro–Wilk test. Accordingly, continuous variables are presented either as mean and standard deviation or as median and range, as appropriate. Group comparison of categorical variables was performed using the Chi-square test (followed by post-hoc testing with Bonferroni corrected *p* values) or the Fisher’s exact test, as appropriate. The Fisher’s exact test was used if the expected count of a cell was less than five. For group comparison of continuous variables, the Mann–Whitney *U* test or the unpaired *t* test was applied. SPSS software version 26.0 (IBM-SPSS, New York, USA) was used for statistical analysis and the level of significance was set at *p* < 0.05.

## Results

A total of 102 patients with a median age of 24 years (range 13–58 years) at the time of the index procedure (first revision ACL-R) were included in this study. The single ACL graft failure group consisted of 58 patients with a median follow-up since the index procedure of 29 months (range 12–124 months), while the multiple ACL graft failures group consisted of 44 patients with a median follow-up of 85 months (range 12–272 months). The follow-up time from the index procedure was statistically significantly longer for the multiple ACL graft failures group compared to the single ACL graft failure group (*p* < 0.001). A detailed summary of the demographic, surgical, and radiographic data is shown in Table 1.

**Table 1:** Demographic, surgical, and radiographic data

Variable	Single ACL graft failure	Multiple ACL graft failures	*p* value
Number of patients, *n*	58	44	–
Age at first revision ACL-R,^a^ (years)	22.5 (13–58)	24.0 (15–49)	n.s
Follow-up since first revision ACL-R,^a^ (months)	29.0 (12–124)	85.0 (12–272)	< 0.001*
Age at primary ACL-R,^a^ (years)	17.5 (12–53)	17.0 (12–34)	n.s
Primary ACL-R to first graft failure,^a^ (months)	18.0 (0–300)	16.5 (1–275)	n.s
Primary ACL-R to first revision ACL-R, (months) ^a^	23.0 (4–308)	26.5 (2–279)	n.s
BMI, (kg/m^2^)	27.0 ± 5.1 (20.5–42.8)	27.4 ± 5.3 (19.6–40.5)	n.s
Males, *n* (%)	29 (50%)	24 (55%)	n.s
Right knee, *n* (%)	27 (47%)	15 (34%)	n.s
Graft primary ACL-R			n.s
Hamstring tendon, *n* (%)	21 (36%)	22 (50%)	
Quadriceps tendon, *n* (%)	3 (5%)	1 (2%)	
Allograft, *n* (%)	16 (28%)	10 (23%)	
N/A, *n* (%)	1 (2%)	0 (0%)	
Graft first revision ACL-R			< 0.001*
Hamstring tendon, *n* (%)	1 (2%)	4 (9%)	
Quadriceps tendon, *n* (%)	32 (55%)	5 (11%)	
Bone-patellar tendon-bone, *n* (%)	7 (12%)	6 (14%)	
Allograft, *n* (%)	18 (31%)	29 (66%)	
Injury mechanism first graft failure			< 0.05*
Atraumatic, *n* (%)	13 (22%)	20 (45%)	
Traumatic non-contact, *n* (%)	41 (71%)	20 (45%)	
Traumatic contact, *n* (%)	4 (7%)	4 (9%)	
Meniscus surgery at first revision ACL-R, *n* (%)	49 (84%)	42 (95%)	n.s
Concomitant surgical procedure at first revision ACL-R			n.s
None, *n* (%)	44 (76%)	39 (89%)	
Meniscal allograft transplantation, *n* (%)	1 (2%)	3 (7%)	
Lateral extra-articular tenodesis, *n* (%)	6 (10%)	1 (2%)	
Osteotomy, *n* (%)	2 (3%)	0 (0%)	
Cartilage surgery, *n* (%)	5 (9%)	1 (2%)	
Contralateral ACL injury	14 (24%)	6 (14%)	n.s
Kellgren-Lawrence Scale			n.s
Grade 0, *n* (%)	21 (36%)	10 (23%)	
Grade 1, *n* (%)	18 (31%)	11 (25%)	
Grade 2, *n* (%)	14 (24%)	11 (25%)	
Grade 3, *n* (%)	5 (9%)	11 (25%)	
Grade 4, *n* (%)	0 (0%)	1 (2%)	
Lateral femoral condyle ratio, (−)^b^	0.65 ± 0.04 (0.56–0.73)	0.65 ± 0.04 (0.60–0.75)	n.s
Medial posterior tibial slope, (°)^b^	9 ± 3 (3–18)	12 ± 3 (6–17)	< 0.001*

Categorical variables are presented as count (percentage of the corresponding group). Continuous variables are presented as mean ± standard deviation (range), unless otherwise noted

ACL, anterior cruciate ligament; ACL-R, anterior cruciate ligament reconstruction; BMI, body mass index; N/A, not available; n.s., non-significant

* Statistically significant difference (*p* < 0.05)

^a^Median (range)

^b^Data available for 98 patients (4 patients had to be excluded because of > 6 mm posterior femoral condyle overlap)

**Fig. 3:**
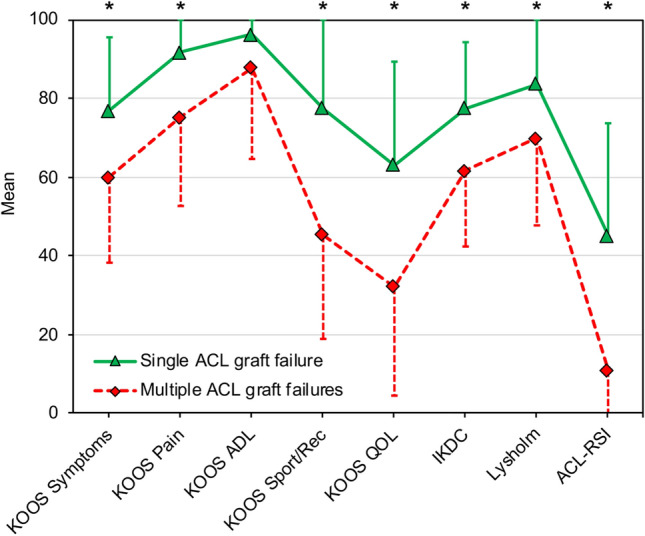
Patient-reported outcome scores with a maximum score of 100 points. Indicators (triangle, rhombus) represent mean values. Error bars represent standard deviation. ACL-RSI, ACL-Return to Sport after Injury Scale; ADL, activities of daily living; IKDC, International Knee Documentation Committee subjective knee form; KOOS, Knee Injury and Osteoarthritis Outcome Score; Sport/Rec, sport and recreation function; QOL, knee-related quality of life; *, statistically significant difference (*p* < 0.05)

Group comparison revealed that statistically significantly more patients underwent first revision ACL-R using quadriceps tendon autograft in the single ACL graft failure group compared to the multiple ACL graft failures group (32 (55%) vs. 5 (11%), *p* < 0.001). Moreover, statistically significantly less allografts were used for first revision ACL-R in the single ACL graft failure group than in the multiple ACL graft failures group (18 (31%) vs. 29 (66%), *p* < 0.001). With respect to radiographic characteristics, multiple ACL graft failures were associated with a statistically significantly higher (steeper) medial PTS than single ACL graft failures (12 ± 3° vs. 9 ± 3°, *p* < 0.001). Although only available for 42 patients (41% of the study group) statistically significantly worse PROs were observed in patients with multiple ACL graft failures compared to patients with single ACL graft failures on all scores collected except for the KOOS subscale “activities of daily living” (Table 2, Fig. 3).

**Table 2:** Patient-reported outcome scores

Variable^a^	Single ACL graft failure	Multiple ACL graft failures	*p* value
IKDC			
KOOS	77.4 ± 16.8 (32.2–100)	61.7 ± 19.3 (17.2–95.4)	< 0.05*
Symptoms	76.8 ± 18.7 (28.6–100)	59.9 ± 21.5 (17.9–92.9)	< 0.05*
Pain	91.8 ± 10.0 (55.6–100)	75.2 ± 22.4 (11.1–100)	< 0.05*
ADL	96.3 ± 6.0 (76.5–100)	87.7 ± 23.0 (16.2–100)	n.s
Sport/Rec	77.5 ± 24.7 (10.0–100)	45.4 ± 26.7 (0–100)	< 0.05*
QOL	63.0 ± 26.5 (0–100)	32.2 ± 27.7 (0–93.8)	< 0.05*
Lysholm Score	83.6 ± 16.6 (25.0–100)	69.9 ± 22.1 (14.0–95.0)	< 0.05*
Tegner Activity Scale^b^	6 (2–10)	4 (0–7)	< 0.05*
ACL-RSI	45.1 ± 28.6 (0–99.1)	11.0 ± 18.8 (0–66.7)	< 0.001*
VAS (pain)	0.9 ± 1.6 (0–6)	2.0 ± 2.4 (0–8)	< 0.05*

Continuous variables are presented as mean ± standard deviation (range), unless otherwise noted

ACL, anterior cruciate ligament; ACL-RSI, ACL-Return to Sport after Injury Scale; ADL, activities of daily living; IKDC, International Knee Documentation Committee subjective knee form; KOOS, Knee Injury and Osteoarthritis Outcome Score; n.s., non-significant; Sport/Rec, sport and recreation function; QOL, knee-related quality of life; VAS, visual analogue scale

*Statistically significant difference (*p* < 0.05)

^a^Data available for 42 patients (41% of study group)

^b^Median (range)

A total of 93 subsequent surgical procedures were performed after the index procedure. In the multiple ACL graft failures group, statistically significantly more patients underwent subsequent surgical procedures compared to the single ACL graft failure group (32 (73%) vs. 8 (14%), *p* < 0.001). Similarly, significantly more patients had complications in the multiple ACL graft failures group than in the single ACL graft failure group (20 (45%) vs. 10 (17%), *p* < 0.05), resulting in a total of 37 complications. A detailed summary of the subsequent surgical procedures performed and the complications observed is shown in Table 3.

**Table 3:** Subsequent surgical procedures and complications

Variable	Single ACL graft failure	Multiple ACL graft failures	*p* value
Subsequent surgical procedure, n (%)	8 (14%)	32 (73%)	< 0.001*
Number of subsequent surgical procedures (per patient)	1.1 ± 0.4 (1–2)	2.0 ± 1.2 (1–7)	< 0.05*
Type of subsequent surgical procedures^a^			< 0.001*
Revision ACL-R, *n* (%)	0 (0%)	35 (42%)	
Hardware removal, *n* (%)	2 (22%)	8 (10%)	
Irrigation/Debridement, *n* (%)	4 (44%)	4 (5%)	
Manipulation under anesthesia, *n* (%)	1 (11%)	2 (2%)	
Meniscus surgery, *n* (%)	2 (22%)	3 (4%)	
Osteotomy, *n* (%)	0 (0%)	9 (11%)	
Cartilage surgery, *n* (%)	0 (0%)	6 (7%)	
Meniscal allograft transplantation, *n* (%)	0 (0%)	13 (15%)	
Lateral extra-articular tenodesis, *n* (%)	0 (0%)	4 (5%)	
Complication, *n* (%)	10 (17%)	20 (45%)	< 0.05*
Number of complications (per patient)	1.1 ± 0.3 (1–2)	1.3 ± 0.5 (1–2)	n.s
Type of complications^b^			n.s
Symptomatic hardware, *n* (%)	2 (18%)	8 (31%)	
Surgical site infection, n (%)	3 (27%)	2 (8%)	
Meniscus tear, *n* (%)	4 (36%)	7 (27%)	
Cartilage lesion, *n* (%)	0 (0%)	6 (23%)	
Knee stiffness, *n* (%)	1 (9%)	3 (12%)	
Symptomatic baker cyst, *n* (%)	1 (9%)	0 (0%)	

Categorical variables are presented as count (percentage of the corresponding group). Continuous variables are presented as mean ± standard deviation (range)

ACL, anterior cruciate ligament; ACL-R, anterior cruciate ligament reconstruction; n.s., non-significant

^a^One patient in the single ACL graft failure group underwent 2 subsequent surgical procedures, and 12, 9, 5, and 1 patients in the multiple ACL graft failures group underwent 2, 3, 4, and 7 subsequent surgical procedures, respectively

^b^One patient in the single ACL graft failure group and 6 patients in the multiple ACL graft failures group experienced two complications

*Statistically significant difference (*p* < 0.05)

## Discussion

The most important finding of this study was that compared to patients with single ACL graft failure patients with multiple ACL graft failures were associated with worse PROs, higher medial PTS (12° vs. 9°), less quadriceps tendon autograft use (11% vs. 55%), and more allograft use (66% vs. 31%).

Similar to primary ACL-R, graft choices for revision ACL-R remain a highly debated topic subjected to many controversies [2–4]. The Multicenter ACL Revision Study (MARS) group has shown that the graft choice affects PROs and failure rates in revision ACL-R [2]. More precisely, patients undergoing revision ACL-R using autografts have been shown to be almost three times less likely to sustain a subsequent graft failure compared to patients undergoing allograft revision ACL-R [2]. Similar results have been shown by another study, which demonstrated a significantly higher Lysholm Score at a mean time of 53 months after revision ACL-R when autografts were used compared to allografts [4]. In addition, the failure rate after revision ACL-R was higher for allografts than for autografts (27% vs. 11%), although not statistically significant [4]. Accordingly, it is evident that autografts are superior to allografts in revision ACL-R, but no final recommendation for the type of autograft has yet been made [2, 4]. The present study showed that in patients with a single ACL graft failure, quadriceps tendon autografts were significantly more often used in first revision ACL-R than in patients with multiple ACL graft failures, where allografts were predominant. The versatility of the quadriceps tendon in ACL-R has been demonstrated in numerous studies and is further supported by the results of this study [9, 23, 24]. While the use of the quadriceps tendon, either with or without an attached patellar bone block, has been established and accepted as a viable graft option for primary ACL-R [9, 23, 25], there are only few reports on the use of the quadriceps tendon as a graft for revision ACL-R [26, 27]. In one study, comparing ipsilateral quadriceps and contralateral hamstring tendon autografts for revision ACL-R, no difference with respect to PROs, instrumented laxity testing, and objective knee evaluation could be observed between the two graft options in 25 (quadriceps tendon) and 26 (hamstring tendon) patients, respectively [27]. However, using the ipsilateral quadriceps tendon had the decisive advantage of avoiding donor site morbidity on the unaffected contralateral limb. Another study showed consistent results with no difference in PROs and anterior–posterior laxity (KT-1000) between patients undergoing revision ACL-R using quadriceps tendon (*n* = 41) or hamstring tendon (*n* = 37) autografts [26]. Notably, after a mean follow-up period of 4.4 years, patients treated with quadriceps tendon revision ACL-R demonstrated significantly less residual rotatory knee laxity based on manual pivot-shift testing [26].

Multiple ACL graft failures are associated with an atraumatic injury mechanism with a gradual onset of recurrent instability [5, 12, 16]. This was confirmed by the present study, in which an atraumatic injury mechanism was significantly more often observed in the group with multiple compared to single ACL graft failures (45% vs. 22%). This might be the result of persistent rotatory knee laxity, which would also explain the high prevalence of meniscal and cartilage lesions in multiple failed ACL-Rs [1, 5, 12, 13, 15, 16, 28, 29]. Since concomitant meniscal and cartilage injuries are associated with the occurrence of OA, it is not surprising that patients with ACL graft failures have significantly more OA compared to patients with an intact ACL graft at long-term follow-up [30]. Although, in this study, more patients with high-grade osteoarthritic changes (Kellgren–Lawrence Scale grade 3 and 4) were identified in the multiple compared to the single ACL graft failures group, no statistical significance was observed.

A higher (steeper) PTS has been associated with ACL injury and rotatory knee laxity, which is even more pronounced in patients with single or multiple ACL graft failures compared to patients with primary ACL injury [5, 13, 31, 32]. This is consistent with the findings of the present study, in which a significantly higher (steeper) medial PTS was observed in patients with multiple compared to single ACL graft failures (12 ± 3° vs. 9 ± 3°). This observation confirms the multifactorial etiology of multiple ACL graft failures, which may lead to a vicious circle involving subsequent surgical procedures, complications, and reduced quality of life. The impact of multiple ACL graft failures on quality of life was demonstrated by significantly worse PROs compared to patients with single ACL graft failures. Given the low average level on the ACL-RSI scale for patients with multiple ACL graft failures, the psychological readiness to return to sport is severely compromised, causing many patients to change the type and level of physical activity [22].

An overall reoperation rate of 11% after revision ACL-R has been reported by the MARS group. Meniscal, revision ACL-R, and cartilage procedures accounted for 27%, 19%, and 17%, respectively, in the MARS cohort [33]. Interestingly, another study by the MARS group demonstrated that patients undergoing more than two revision ACL-Rs are 4.7 times more likely to require subsequent surgery compared to patients undergoing a first-time revision ACL-R [2]. This was also confirmed in the present study, in which patients with multiple ACL graft failures were more likely to undergo subsequent surgery and had significantly more subsequent surgical procedures than patients with a single ACL graft failure.

One limitation of the present study was the significantly longer follow-up period since the first revision ACL-R for patients with multiple compared to single ACL graft failures. Given that many patients already experienced multiple ACL graft failures at the time of first consultation, this was to be expected. Previous studies comparing the results of primary and multiple revision ACL-R may also be subjected to this bias, since the reported follow-up time is usually related to the last revision ACL-R [5, 12]. Comparing the follow-up period since the last revision ACL-R implicates a difference in the follow-up period since the first revision ACL-R, which is the most appropriate index procedure for comparing such patients. Patients in the single ACL graft failure group may experience subsequent ACL graft failures in the future. Therefore, a minimum of 12-month follow-up represents another limitation of this study. In addition, PROs were not available in all patients, which is associated with the retrospective design of this study.

## Conclusions

In this study, multiple ACL graft failures were associated with worse PROs, higher (steeper) PTS, and the use of allografts compared to single ACL graft failures. During the first revision ACL reconstruction, it is recommended to avoid the use of allografts and to consider slope-reducing osteotomies to improve functional outcomes by reducing the risk of multiple ACL graft failures.

## Abbreviations

ACL: Anterior cruciate ligament
ACL-R: Anterior cruciate ligament reconstruction
BMI: Body mass index
ICC: Intraclass correlation coefficient
LFCR: Lateral femoral condyle ratio
MARS: Multicenter anterior cruciate ligament revision study
MRI: Magnetic resonance imaging
OA: Osteoarthritis
PROs: Patient-reported outcomes
PTS: Posterior tibial slope
